# Worksite Physical Activity Barriers and Facilitators: A Qualitative Study Based on the Transtheoretical Model of Change

**DOI:** 10.3389/fpubh.2018.00326

**Published:** 2018-11-15

**Authors:** Jo-Hanna Planchard, Karine Corrion, Lisa Lehmann, Fabienne d'Arripe-Longueville

**Affiliations:** Université Côte d'Azur, LAMHESS, Nice, France

**Keywords:** physical activity, workplace, transtheoretical model, behavior change, stage of change

## Abstract

**Background:** Many of the studies on worksite physical activity (PA) have investigated either the effectiveness of PA programs for employees and the work-related outcomes or health promotion interventions to increase PA. However, studies on barriers and enabling factors for participation are scarce and have generally not been theoretically grounded. The purpose of this qualitative study was to identify worksite PA barriers and facilitators from the perspective of the transtheoretical model of change (TTM).

**Methods:** Thirty employees (15 females and 15 males; M_age_ = 44.70; *SD* = 5.20) were recruited to participate in semi-structured interviews lasting from 60 to 90 min. Participants came from several organizations that offered PA programs and were at different exercise stages of change. They were invited to describe: (a) general information on the place of PA in their daily lives and in the workplace, and the reasons for (b) worksite PA participation or (c) non-participation. The interview transcripts were analyzed both inductively and deductively with reference to the exercise stages of change.

**Results:** Three categories of barriers and facilitators related to physical, psychological and environmental dimensions were identified. For all exercise stages of change combined, psychological and environmental barriers were significantly more reported than physical barriers, whereas physical and psychological facilitators were more cited than environmental facilitators. Further qualitative analysis suggested that these categories differed with the exercise stage of change. At the precontemplative and contemplative stages, all types of barriers predominated (e.g., physical constraints due to the workstation, fear of management disapproval, time constraints). At the preparation stage, physical, and psychological needs emerged in relation to worksite PA (e.g., need to compensate for sedentary work, stress regulation). At the action and maintenance levels, physical, psychological, and environmental facilitators were reported (e.g., enhanced physical condition, workplace well-being, social ties). At the relapse stage, specific life changes or events broke the physically active lifestyle dynamics.

**Conclusion:** This study identified the contribution of different types of worksite PA barriers and facilitators according to the exercise stage of change. The identified facilitators are consistent with the general TTM processes of change, while being specific to the workplace. Practical strategies are discussed.

## Introduction

The international community (1) has recognized the workplace as a suitable site for health promotion and raising awareness of the risk factors for obesity, diabetes, and cardiovascular disease. Physical activity (PA) programs to improve employee health have been implemented in many organizations, and research on this subject has correspondingly intensified. Three main categories of studies can be identified in the literature. The first category pertains to the types of worksite PA interventions that have been implemented. A recent systematic meta-review by Jirathananuwat and Pongpirul (2) classified and described interventions to promote workplace PA based on the evidence from systematic reviews and meta-analyses. Using the PRECEDE-PROCEED model, the interventions were classified into predisposing, enabling, reinforcing, environment, and policy domains of focus. Of the 48 interventions identified, 22 (46%) focused on predisposing employees to have more PA (information delivery and training programs) and 17 (35%) focused on enabling them to do so (instrument resources and health service facilities). The reinforcing approaches included incentives and social support, whereas the remaining interventions targeted environmental development and policy regulation.

The second category of studies examines the effects of PA programs and has revealed quite inconsistent findings to date. The meta-analysis by Conn et al. (3) indicated that workplace PA interventions can improve employee health (i.e., PA behavior, fitness, lipids, anthropometric measures, and job stress). More recent studies have also suggested that workplace interventions that are compatible with productive work (i.e., alternative workstations, interventions promoting stair use, and personalized behavioral interventions) reduce sedentarity and increase PA behaviors at work, with some of the interventions (i.e., multi-component and environmental strategies) positively influencing these behaviors in all aspects of daily life (4, 5). Nevertheless, the evidence has been inconsistent regarding the impact of these programs on employee productivity, including measures like absenteeism, employee turnover, and job satisfaction (6, 7), and the cost-effectiveness for employers (8). In addition, the long-term effects of PA interventions remain to be established.

The third category of research examines the factors that determine participation in workplace PA programs. A few qualitative studies have explored perceived barriers and/or facilitators of workplace PA using individual semi-structured interviews (9, 10) and focus groups (11). The results have consistently shown that the main barriers are physical limitations due to pain and weakness, lack of motivation, lack of time, and work commitment, whereas the strongest motivators are family relationships, social support and perceived health benefits (e.g., better health management). Other studies have pointed out that the barriers emerge from an interaction between management, employees, and intervention characteristics, thus emphasizing the importance of organizational support strategies (12–14). The quantitative studies have demonstrated that sociodemographic factors like gender and the type of intervention (i.e., multi-behavior) are significant factors of participation (15). For example, Beck et al. (16) reported that white, female, non-union staff and employees seeking preventive care were the most likely to participate in preventive programs. A few studies have examined the barriers and motivators of worksite PA from the perspective of sociocognitive models. Keller et al. (17) reported an increase in self-efficacy, planning and PA following a workplace intervention and showed that planning was consistently associated with subsequent PA. Hadgraft et al. (18) suggested that strategies aimed at increasing employees' perceived control and self-efficacy over their sitting time might be helpful components of workplace interventions, although they only partially explained the variation in reduced workplace sitting.

Deeper insight into the perceived barriers and facilitators of worksite PA is needed at this point and this might be best accomplished through studies based on theoretical models of behavior change. An important factor that can explain individual variability among employees is the exercise stage of change. The transtheoretical model (TTM), which deals with behavior change through a temporal dimension of readiness to change (19), has been applied to several health behaviors, and it was also found useful for understanding the mechanisms by which people become physically active (20, 21). PA behavior change occurs through a series of five stages (22, 23): (a) precontemplation (i.e., no intention of becoming physically active and awareness of the problems associated with this behavior), (b) contemplation (i.e., awareness of the negative effects of inactivity with intention to start practicing PA), (c) preparation (i.e., making small changes in behavior—joining a gym, for example—but still not meeting a criterion for physical activity), (d) action (i.e., meeting a criterion of physical activity, but only recently—usually within the past 6 months), and (e) maintenance (i.e., meeting a criterion for physical activity for 6 months or longer).

Decisional balance, which can be defined as a balance in the perceived advantages and disadvantages of change, is one of the factors hypothesized to mediate the change process. The perception of the advantages associated with PA (i.e., facilitators) is strong in the last two stages (i.e., action and maintenance), whereas the perception of the disadvantages (i.e., barriers) is strong in the early stages (i.e., precontemplation and contemplation) (24). Preparation is the stage at which the potential gains are in balance with the perceived losses. Stage progression follows a somewhat cyclical pattern, with individuals progressing and regressing through the stages. The perceived barriers and facilitators are important for predicting the transitions between precontemplation, contemplation, and preparation, but less so for action and maintenance (21).

Many worksite PA studies have focused on the types and effectiveness of PA programs for employees and the work-related outcomes. Research on the barriers and enabling factors of participation is scarce, however, and in general has not been theoretically grounded. It seems likely that the TTM would help capture the PA stages of change of employees, but it has never been applied to worksite PA. The purpose of this qualitative study was thus to provide a comprehensive understanding of worksite PA barriers and facilitators from the perspective of the Transtheoretical Model of change (TTM).

## Methodology

### Study design and setting

This study used qualitative methods involving individual interviews. The interview guide was developed on the basis of the literature on health (24), worksite (15), and exercise psychology (25). The research team was familiar with this literature and qualitative methods. The interviews were semi-directive, with semi-structured questions (26, 27). The procedure had three phases. First, following authorization from the different organizations, an initial visit was arranged to explain the nature and goal of the investigation and distribute consent forms. Signed informed consent was obtained prior to conducting the interviews. Second, the first author conducted a pilot interview with two employees (one male and one female). These interviews helped to adjust the interview guide and ensure the flow of the interviews, respecting the principles of sympathetic understanding (28) and neutrality (27). Third, the individual semi-structured interviews were conducted and lasted an average of 40 min. Each interview was recorded in a private room with no distractions. Written notes were used to facilitate follow-up questions. Confidentiality was ensured and the following coding system was adopted: T1 to T30. Once transcribed, the verbatim interviews were given to the participants so that they could check the content and quality of the transcript. This entailed a few minor changes.

During the interviews, the participants were invited to provide information about: (a) PA practice in their daily lives and (b) the reasons for worksite PA participation or (c) the reasons for worksite PA non-participation. The first part included general information on the types, forms and locations of PA in the employees' daily lives (e.g., *What type of PA do you do in your free time?*). The second part aimed to identify the perceived barriers (arguments against) and facilitators (arguments for) of PA practice in daily life (e.g., *What elements contribute to your regular PA practice?*). The third part was devoted to the perceptions of the barriers and facilitators of workplace PA practice [e.g., *What are the reasons for practicing (or not practicing) physical activity in the workplace?*]. Finally, in part three, questions about the worksite PA offer were raised: *What do you think about the PA offer that is available? What are the advantages or disadvantages of this offer?*

### Study sample

Thirty French employees (15 females and 15 males; M_age_ = 44.70; *SD* = 5.20) were recruited. In order to have a variety of workplace profiles, participants were recruited in public or private organizations offering PA programs: (a) 10 employees from a public university, (b) 10 employees from a hospital, and (c) 10 employees from private companies (see Table 1). They were predominantly white and educated. All the participants were at different exercise stages of change (29). All gave signed informed consent before engaging in the study.

**Table 1:** Socio-demographic and workplace characteristics among participants (*n* = 30).

**Variables**	**Number**
**GENDER**	
Women	15
Men	15
**AGE (YEARS)**	
20–40	13
40–60	17
**JOB CHARACTERISTICS**	
Administrative staff	11
Doctors/Nurses/Professors/Teachers/Engineers	10
Lab technicians/workers/Assistants	9
**EDUCATION**	
Elementary school	0
Middle school	2
High school	10
College	18
**MARITAL STATUS**	
Single	4
Married or living with someone	15
Separated or divorced	9
Widower	2
**STAGE OF CHANGE**	
Precontemplation/contemplation	8
Preparation	6
Action/maintenance	11
Relapse	5

### Data analysis

The interviews were transcribed *verbatim*. The interviews were stopped when saturation was reached, which was the point at which no new themes or information emerged (30). To ensure that the determined codes and categories were embodied in rather than forced on the data (31), we adopted both inductive and deductive content analyses for the interview transcripts (32). Deductive content analysis was based on the categories (physical, psychological, and environmental) of barriers and facilitators in relation to the exercise stages of change. Two researchers (the first and last authors) independently coded the transcripts. They identified and grouped meaning units (MUs) into subcategories within the main categories. The researchers discussed the categorization until consensus was reached. Then, two other researchers, the second and third authors, considered as disinterested peers (i.e., blind to the analysis and purpose of the study), were invited to verify the encodings and interpretations of the data (30). Their analyses were up to 92% in agreement with those of the first two researchers, which is considered high (33, 34). Last, a binomial exact test was used to compare a proportion with an expected value, all exercise stages of change combined. Results were considered significant at *p* < 0.05.

## Results

All the transcribed interviews were analyzed line by line and this content analysis identified 623 MUs related to the study purpose: 272 MUs were related to the perceived barriers to workplace PA and 351 MUs were related to the perceived facilitators.

### Perceived barriers and facilitators of worksite PA

Three categories of barriers and facilitators related to the physical, psychological and environmental dimensions were identified (see Table 2). The binomial exact test analysis revealed that the employees cited psychological or environmental barriers significantly more often than physical ones (Binomial = 0.022, *p* = 0.5), but reported physical or psychological facilitators significantly more often than environmental ones (Binomial = 0.001, *p* = 0.5). Of the perceived physical facilitators of PA practice in the workplace, 86% of the employees reported improved fitness. The most often mentioned psychological benefit was awareness of the positive effects of PA on health, reported by 76%. For the environmental facilitators, 53% of the participants mentioned time savings. The most often mentioned physical barrier was the physical constraints specific to the job position (53%), thus limiting the employee's commitment to PA in the workplace. The low acceptability of PA in the organization's norms was the most frequently mentioned psychological barrier, noted by 60% of the employees. For the environmental barriers, inadequate program supervision was noted by 56%.

**Table 2:** Perceived barriers to and facilitators of worksite PA practice at workplace.

	**Types**	**Number of MUs**	**Number of employees**	**Characteristics of barriers and facilitators**
	Physical	230 MUs (65.5%)	26 21 12 9	– Improved physical fitness – Weight loss – Disease prevention – Improved sleep
Perceived facilitators of PA practice at the workplace (351 MUs; 56.3%)	Psychological	90 MUs (25.7%)	24 12 10 7 6	– Awareness of positive effects of PA on health – Improved cognitive efficiency at work – Improved social self-esteem at work – Improved social ties in the organization – Improved psychological well-being and less work stress
	Environmental	31 MUs (8.8%)	16 8	– Time savings (remain onsite) – Financial advantages
	Physical	82 MUs (30%)	16 5 4	– Physical constraints of workstation – Physical restrictions of workstation – Physical limitations (fatigue, pain due to exercise)
Perceived barriers to PA practice at the workplace (272 MUs; 43.7%)	Psychological	83 MUs (30%)	18 12 8 7	– Perceived low acceptance of PA according to organization norms – Fear of reduced productivity – Fear of management disapproval – Fear of physical discomfort (sweating)
	Environmental	107 MUs (40%)	17 11 9 8	– Inadequate offer of PA – Inadequate equipment available – Inconvenient hours – No showers or changing rooms

*N = 30; MUs, Meaning Unit*.

### Worksite physical activity barriers and facilitators according to the participants' stage of change

The perceptions of barriers and facilitators changed over the course of the stages of change (i.e., precontemplation and contemplation, preparation, action/maintenance, and relapse). The results are shown in Table 3.

**Table 3:** Worksite physical activity barriers and facilitators according to the participants' stage of change.

**Stage of change**	**Physical domain**		**Psychological domain**		**Environmental domain**	
	**Barriers**	**Facilitators**	**Barriers**	**Facilitators**	**Barriers**	**Facilitators**
PC/C (*n* = 8)	– Physical constraints linked to job position (*n* = 5) – Physical limitations (fatigue, pain) (*n* = 4) – Restrictions of job position (*n* = 2)		– Fear of management disapproval (*n* = 3) – PA not a priority for the employee (*n* = 2)	– Awareness of positive effects of PA for health (*n* = 8)	– Work overload /not enough time (*n* = 6) – Inconvenient PA hours (*n* = 4)	
PR (*n* = 6)	– Physical limitations (fatigue, pain) (*n* = 4)	– Need to physically compensate for sedentary work (*n* = 5) – Need to lose weight (*n* = 4) – Need to have a healthier lifestyle (*n* = 2)	– Fear of lower productivity (*n* = 4) – Fear of physical discomfort (effort; muscle aches; sweating) (*n* = 2)	– Need to handle work-related stress (*n* = 4) – Awareness of PA benefits for health (*n* = 3) – Perception of management approval (*n* = 2) – Motivational contagion between employees (*n* = 2)	– Work overload/not enough time (*n* = 3) – Inadequate program and/or framework proposed (*n* = 2) – Poorly adapted equipment made available (*n* = 2) – No showers or changing rooms available (*n* = 1)	– Time savings (remain onsite) (*n* = 4) – Financial advantage (*n* = 3)
A/M (*n* = 11)		– Improved physical fitness (*n* = 10) – Weight loss (*n* = 8) – Improved sleep (*n* = 5) – Healthier lifestyle (eating better; stopped smoking) (*n* = 2)		– Reduced work-related stress (*n* = 7) – Improved psychological well-being (*n* = 6) – Improved social self-esteem at work (*n* = 5) – Improved cognitive efficiency on the job (*n* = 3) – Improved social ties in the organization (*n* = 2) – Management approval (*n* = 2)	– Work overload / not enough time (*n* = 4)	– Time savings (remain onsite) (*n* = 8) – Financial advantage (*n* = 7)
R (*n* = 5)	– PA-related injury (*n* = 2) – Work accident (*n* = 1) – Sick leave (*n* = 1)		–Major conflict with a manager or colleague (*n* = 3)		– PA hours changed (*n* = 2) – Program or framework changed (*n* = 2) – New family constraints (*n* = 1)	

*PC/C, Precontemplation/Contemplation stages; PR, Preparation stages; A/M, Action/Maintenance stage; R, Relapse*.

#### Precontemplative and contemplative stages

The employees in these stages were physically inactive and had little interest in PA in general and at the workplace in particular. They mostly mentioned barriers to practice compared to facilitators.

##### Physical domain

The main physical barriers were: (a) the physical constraints of the job, (b) personal physical limitations, and (c) job restrictions. The physical constraints were characterized by the activities inherent to the job, as illustrated in this excerpt: “*I help patients to get up and wash every day – I already do PA at work*” (M25). PA was also seen as potentially causing physical limitations, such as fatigue or pain, as noted in the following excerpt: “*I'm tired enough with my daily work, so I'm not going to tire myself out any further*” (M12).

##### Psychological domain

Although all participants were aware of the health benefits of PA, several barriers could be identified at the psychological level. First, the employees were sensitive to the degree of PA acceptability within their organization and feared the disapproval of management, as expressed by these participants: “*The employer takes a dim view of PA*” (M4) with PA being seen as synonymous with “*leisure time and amusement*” (F8). In addition, many expressed the conviction that workplace PA was not a priority and for a variety of reasons. Some believed that work was not the right place for PA and they preferred to keep their autonomy for this practice “*out of respect for my private life”* (F11) and “*not [to] be seen by the other employees*” (M15); others thought it would hinder their productivity, as evidenced by this excerpt: “*Right now I have other priorities at work; I have objectives I want to reach*” (M9).

##### Environmental domain

Environmental barriers were mentioned such as a heavy workload and time constraints.

#### Preparation stage

In the preparation stage, the employees were preparing for change, making the actual decision to do so. They expressed a balance between perceived barriers and facilitators.

##### Physical domain

Although limitations were still mentioned (e.g., fatigue, musculoskeletal disorders, joint pain), several facilitators were identified: (a) the need to physically compensate for a sedentary job, (b) the need to lose weight, and (c) the need to improve one's lifestyle, as respectively illustrated in the following excerpts: “*I sit in front of my screen for 8 hours a day; I feel a need to move, to do something athletic*” (M12). “*Since I've been working here, I've gained 20 pounds and my doctor told me to lose weight to protect my knees and back”* (M6).

##### Psychological domain

Several barriers were still mentioned, such as the fear of being unproductive on the job and the fear of physical discomfort, notably muscle aches and sweating. On the other hand, several types of psychological facilitators were noted in addition to the awareness of the perceived health benefits of PA mentioned in the earlier stages. The main facilitator was the possibility of managing the work stress generated by aggressiveness both within and outside of the organization, as illustrated by the following excerpts: “*When I'm involved in a physical activity, I feel less stressed out*” (M7). “*PA helps me to react better to the aggressiveness of patients and their families*” (F1). Many of the participants said that management's approval of this practice was very important, as illustrated by this excerpt: “*If this proposal is supported by top management, I'd be interested in trying it*” (F3). Last, motivational contagion from the group involved in the PA program was mentioned by many as a facilitating factor in deciding to begin workplace PA (e.g., positive group dynamics, social ties, conviviality): “*Doing this with my work colleagues is fun and makes me want to continue*” (M10).

##### Environmental domain

In addition to the workload and lack of time expressed in previous stages, the following environmental barriers were mentioned: (a) an inadequate program and/or framework, (b) inadequate equipment, and (c) no accessibility to showers and/or changing rooms. These excerpts illustrate some of these barriers: “*We would like to have a personalized coach*” (M14). “*There are no showers or changing rooms at the workplace*” (F2). Two categories of environmental facilitators were identified: (a) time savings and (b) financial benefits, as expressed by this employee: “*It's not expensive compared to a club*” (M27).

#### Action and maintenance stages

In these stages, the change was real and observable. Barriers were no longer brought up and the employees spoke only of the facilitators, even though perceptions of a work overload and a lack of time persisted.

##### Physical domain

The main physical facilitators were the improvement in overall fitness and weight loss, as these remarks show: “*Before, I got breathless when I took the stairs; since I've been practicing PA, I feel the difference*” (F25). “*Since I started PA, I've lost weight and I feel better*” (F24). “*I sleep better at night and I now have a healthy lifestyle*” (M7).

##### Psychological domain

Six types of psychological facilitators were identified: (a) reduced work stress (“*I'm working in better conditions and I feel more relaxed since I started*,” M22), (b) improved psychological well-being, (c) improved social self-esteem at work (“*PA practice improves the image I have in my work compared to inactive colleagues*,” M26)., (d) improved cognitive efficiency (“*We're more efficient at work since we started PA*,” F28)., (e) improved social ties within the organization (“*It's a nice moment for coming together and sharing*” (F11) with “*a really positive group dynamic*,” M1)., and (f) the approval of management (“*There is a real corporate culture around sport. We feel that it is supported and approved of. So it encourages us to participate without guilt!*” F3).

#### Relapse stage

In the relapse stage, the employees cited only barriers.

##### Physical domain

Three physical barriers were identified that could occur at any time: (a) injury during PA practice, (b) a work accident, and (c) a sick leave. The following excerpt illustrates this type of physical barrier to practice: “*I've had to stop my physical activity since my work accident*” (M24).

##### Psychological domain

A conflict with a supervisor or colleague was a psychological barrier to PA practice, as this quote indicates: “*I don't participate anymore because I don't want to run into this colleague at the PA sessions*” (F15).

##### Environmental domain

Last, environmental changes could be barriers to daily practice at this stage, such as: (a) a change in session schedules, (b) a change in the framework and/or program, and (c) new family constraints.

## Discussion

The aim of the present study was to better understand the barriers to and facilitators of worksite PA from the perspective of the transtheoretical model of behavior change. The analysis of the qualitative data collected during the semi-structured interviews identified three categories of barriers and facilitators related to the physical, psychological and environmental domains, and the repartition differed with the participants' exercise stages of change.

An important finding is that the PA barriers and facilitators that emerged were multidimensional and shared similarities with those of other contexts and populations, while also showing specific workplace-related characteristics. The most frequently mentioned barriers concerned the physical constraints related to the workstation, the low acceptability of PA in the organization's norms, the fear of lower productivity, and the inadequacy of the equipment or supervision. The main identified facilitators were improved physical fitness and weight loss (physical dimension); awareness of the positive effects of PA on health, improved cognitive effectiveness at work, and improved social self-esteem at work (psychological dimension); and time and money savings (environmental dimension). A part of these results is consistent with the barriers and facilitators previously identified in the general population or vulnerable populations. For instance, the barriers related to physical constraints such as fatigue or pain, or the inadequacy of equipment or supervision, appear as obstacles widely shared by the general population (35, 36) including employees (9), older people (37), and people with chronic diseases (38). In the same vein, facilitators such as improved physical fitness and weight loss (physical dimension), awareness of the PA benefits to health, and time and money savings appear as common enablers of PA in the literature (35, 39). However, factors such as the low acceptability of PA in the organization's norms (and therefore fear of management disapproval) and fear of lowered productivity appear as factors pertaining to professional activity and its context. From this point of view, our results provide support to previous work showing the role of organizational-support strategies (40). Similarly, improved cognitive effectiveness and social self-esteem at work appear as worksite-related facilitators of PA already outlined in the workplace literature (6).

One strength of this study is that it highlights the shifting distribution of these three categories of PA barriers and facilitators according to the employees' stages of change based on the TTM framework (22, 23). The precontemplative and contemplative stages were characterized by all types of PA barriers (e.g., physical constraints due to the job position; fear of management disapproval; lack of time), although awareness of PA benefits for health was observed for all participants. At the preparation stage, physical and psychological needs related to worksite PA (e.g., need to compensate for sedentary work, stress regulation, and time savings) emerged. At this stage, there was a balance between perceived PA facilitators (e.g., need to lose weight or improve one's lifestyle; motivational contagion between employees) and barriers (e.g., fear of lowered productivity or physical discomfort; inadequacy of the program, coaching, and/or equipment). At the action and maintenance levels, physical, psychological, and environmental facilitators were mainly reported (e.g., enhanced physical condition, workplace well-being, social ties at work) while the barriers were minor (e.g., workload). Finally, at the relapse stage, specific life changes or events (e.g., accident, conflict, or a change in schedules) could break the physically active lifestyle dynamics. These events were able to cause a relapse in employee behaviors and reverse the decisional balance toward perceived barriers. These results enrich the existing literature on physical activity at the workplace based on the TTM model (20, 29) by providing a qualitative understanding of the stages of change dynamics.

The strengths of this work, as with any study, should be considered in light of its limitations. First, the generalizability of our findings may be questioned given the limited sample size. Although we used purposive sampling to ensure a variety of workplaces and professional activities, additional interviews in other professional contexts would need to be conducted to enrich our results. Moreover, the participants were interviewed about a potentially sensitive topic and may have given socially desirable answers. To address this in future research, observational data could be added to assess the consistency of different data sources (41). Selection bias could also have resulted from a non-response. It may be that the employees who participated in this study were more engaged in addressing lifestyle issues than those who did not participate. Finally, due to the limited time of those who participated, it was difficult to conduct in-depth interviews of 60 min. Therefore, our interviews lasted an average of 40 min. Although we found a great variety of answers and saturation was reached or almost reached in the last interviews, more time might have favored the emergence of new subthemes.

The results of this study suggest several practical implications. First, managers and public health practitioners who want to promote PA in the workplace will have to take into account their employees' stages of change and the multidimensional nature of the barriers and facilitators allowing them to evolve. Second, our results suggest that promotion strategies will have to attempt to resolve some paradoxes. Although the employees appeared globally aware of the positive effects of PA for health, the workplace did not seem to be an adapted context for all of them. Specifically, the fear of lowered productivity might have prevented some of them from engaging in worksite PA, even though those who did so reported better cognitive efficiency at work after exercising. Furthermore, the major psychological barrier, which was the low acceptability of PA in the organization's norms, indicates the importance of ensuring that worksite PA becomes a strategic priority and that PA interventions are smoothly integrated into the organizational routines. Finally, the wide range of the employees' needs suggests that the PA offers should be as individualized as possible and particularly adapted to the constraints of workstations and motivational profiles.

## Conclusion

This study offers an original qualitative insight into the multidimensionality of PA barriers to and facilitators of worksite PA. For all exercise stages of change combined, psychological and environmental barriers were significantly more reported than physical barriers, whereas physical and psychological facilitators were more cited than environmental facilitators. However, framing this study within the TTM allowed us to capture their dynamics according to the participants' exercise stages of change. Pursuit of such work should be beneficial to public health practitioners and organizations for the design of optimal PA strategies, which should offer PA programs and intervention contexts based on the barriers and facilitators characteristics of each exercise stage of change. Future research based on the TTM should specify the most favorable workplace interventions to help employees moving from one stage to another and should examine the effects of programs in light of these stages of changes along with motivational profiles and workstation constraints.

## Ethics statement

The protocol of this study was approved by the local committee of The Université Côte d'Azur.

## Author contributions

J-HP oversaw collection of the data, contributed to the analysis and interpretation of the results, and drafted the results' section of the manuscript. KC contributed to the analysis and interpretation of the results, and contributed to the writing of the manuscript. LL contributed to the interpretation of the results and oversaw the writing of the manuscript. FdA-L was responsible for the scientific project and oversaw the collection and analysis of data, contributed to the interpretation of the results, and helped writing the manuscript. All authors have read and approved the final version of the manuscript and agree with the order of presentation of the authors.

### Conflict of interest statement

The authors declare that the research was conducted in the absence of any commercial or financial relationships that could be construed as a potential conflict of interest.
